# ‘Sex Is Not Just about Ovaries.’ Youth Participatory Research on Sexuality Education in The Netherlands

**DOI:** 10.3390/ijerph17228587

**Published:** 2020-11-19

**Authors:** Marianne Cense, Steven de Grauw, Manouk Vermeulen

**Affiliations:** Research Department, Rutgers Netherlands, 3506 GA Utrecht, The Netherlands; stevendegrauw@gmail.com (S.d.G.); m.vermeulen@rutgers.nl (M.V.)

**Keywords:** sexuality education, sexuality, sexual agency, youth, student voice, youth participatory action research, The Netherlands

## Abstract

Young people are not satisfied with the sexuality education they receive in Dutch high schools. They rate their sexuality education as mediocre (5.8 on a scale of one to ten). In cooperation with 17 young peer researchers, we explored what good sexuality education looks like from the point of view of young people. The peer researchers collected data in their own high school, using mixed methods, starting with individual interviews, followed by focus group discussions and Photovoice sessions to get more in-depth views on topics, class atmosphere, and teacher skills. In total, 300 pupils aged 12–18 participated in the research. Our findings demonstrate that young people want more sexuality education, during their whole school career. They want sexuality education to move beyond biological functions, sexually transmitted diseases, and reproduction into issues like dating, online behavior, sexual pleasure, relationships, and sexual coercion. Moreover, pupils want sexual diversity integrated and normalized in all content. One of the main issues is that sexuality education should be given in a safe class atmosphere, which demands sensitivity from the teacher. In addition to the findings of the study, this article reflects on the steps to be taken to realize the changes desired by young people.

## 1. Introduction

Although the Netherlands is seen as a forerunner when it comes to sexuality education [1,2], Dutch high school pupils think the sexuality education they receive can be improved. Young people rated the information they received in school as mediocre (5.8 on a scale of one to ten), as shown by the Dutch survey Sex under the age of 25 [3]. Lesbian, gay, and bisexual youth-rated it even lower with a 5.2. From the survey, we cannot distinguish the reasons for these low ratings, but they clearly raise concerns about the current practice of sexuality education in the Netherlands. This is reflected in an inquiry by the Dutch Inspectorate of Education in 2016 [4], which showed that sexuality education at schools was dependent on the teacher and not delivered on a structural basis, but randomly.

What is considered sexuality education varies across countries and programs? Often, sexuality education is shaped by a narrow understanding of sexuality, focusing on sexual contacts, sexual anatomy, reproduction, birth control, and disease prevention [5]. Our interpretation of sexuality education is based on the World Health Organization European Expert Group on Sexuality Education [6], looking at sexuality education as a lifelong learning process of acquiring information and of forming attitudes, beliefs, and values about relationships, intimacy, and identity. Sexuality education has been mandatory in Dutch primary and secondary schools since 2012. However, the national education curriculum merely says that schools must pay attention to sexual diversity and sexuality, without giving any guidelines on content, frequency, or number of classes. The Dutch constitutional principle of freedom of education creates a barrier for a nationwide implementation of sexuality education, as Dutch schools are free to determine the amount of time, the approach, and the methods they use. Comprehensive Sexuality Education (CSE) is characterized by a positive approach to sexuality that accepts sexual feelings, desire, and pleasure as essential components of young people’s sexuality [5,6]. A comprehensive approach puts sexuality in a wider perspective of personal growth, development, and building up mutually consensual (sexual) contacts and relationships [5]. Dutch evidence-based, comprehensive sexuality education methods are available for use in- and outside of biology, but the implementation of these methods highly depends on contextual factors, such as teacher training, school policy, governing body support, and student response [7]. At present, sexuality education is predominantly taught within a biology framework, linking sexuality to puberty, sexual reproduction, prevention of sexually transmitted diseases (STDs), and unintended pregnancies [8]. Consequently, Dutch pupils often only receive sexuality education from 13 to 15 years as many pupils quit biology in senior grades. 

Sexuality education has frequently been criticized for its failure to meet the needs and hold the interest of young people [9,10,11,12]. This should not be a surprise, as increased efforts to provide young people with sexuality education often emerge from moral panic around higher rates of teenage pregnancy, STDs, or sexting, with the aim to control young people’s sexualities, displaying coherent medical and moral values [13]. Allen’s study amongst young people aged 16–19 in New Zealand showed that content that does not address the questions and issues young people deem important may be dismissed as irrelevant [10]. One of the main issues Allen found was that the content of many sexuality education programs is very protective and directed towards risk management, offering young people very limited space for sexual agency, which does not register with their own sense of self and entitlement and even disempowers them. 

Scholars also pointed out that many sexuality education materials and practices are not tailored to fit the needs of lesbian, gay, bisexual, transgender, and queer/questioning (LGBTQ) youth [14,15,16,17]. The higher rates of dissatisfaction with sexuality education amongst young people who differ from cultural ‘normalcy’, for instance, by being non-heterosexual, can be explained by the link between sexuality education and the dominant sexual culture. Sexuality education runs the risk of reproducing and reinforcing social inequalities and injustice, as it reflects the values, ideas, and stereotypes of the dominant culture [18,19]. Sexuality education may easily reproduce existing sexist, racist, and classist notions of sexuality, thereby ‘projecting a particular message and vision of who and how teens are and should be’ [18] (p. 61). This plays out not only on heteronormativity, but also on gender inequality, classism, and racism [20,21,22,23] and secularism/religious diversity [24,25,26].

Another field of concern that is illuminated by many scholars is the lack of a safe class atmosphere during sexuality education [15,20,27,28,29]. Sexuality is not just the subject of sexuality education; it is present all the time within and outside classrooms. The school is a space where sexuality is played out [28,29]. Krebbekx’ ethnographic study of sexuality education practices in Dutch schools shows how sexuality education classes can be used by young people to reproduce existing popularity hierarchies in class and how the probing by teachers to open up about their experiences can lead to bullying or publicly ’outing’ someone as sexually active [28]. Clearly, creating a safe atmosphere is closely linked to teachers’ pedagogic strategies, which is an area that arises in several studies on what young people voice as a concern in how sexuality education is delivered [10,11,12,13,14,15,30]. A meta-analysis of qualitative studies on sexuality education in ten countries illuminated three main problems [31]. The studies originated from the UK, Ireland, the USA, Australia, New Zealand, Canada, Japan, Iran, Brazil, and Sweden. Firstly, schools take insufficient account of the ‘specialness’ of sex as a topic: ‘[S]ex is a potent subject that can arouse strong emotions, reactions, and feelings (…) yet the prevailing approach within schools appears to be to deny that there is anything exceptional about the topic and to attempt to teach sexuality education in the same way as other subjects’ [31] (p. 4). Secondly, the study suggests that schools struggle to accept that some young people are sexually active, leading to sexuality education content that is out of touch with the lives of sexually active young people. Finally, the evidence indicates that young people dislike having their own teachers deliver sexuality education. Other studies illuminating young people’s perspective put emphasis on the desire for different content in sexuality education: Young people want less information about biological aspects of sexuality, and more explicit and accurate information about gender and sexual diversity, violence in relationships, intimacy, sexual pleasure, and love [10,32]. Studies also highlight that young people not only want classroom-based sexuality education, but also online sexuality education programs, with practical, age-appropriate content in the age range from primary school through to university [33,34]. As sexuality education, sexual culture, and the sexual practices of youth are strongly embedded in the cultural context, we cannot simply transfer the findings of international studies to the Netherlands. This paper explores what good sexuality education looks like from the point of view of Dutch young people.

## 2. Materials and Methods

### 2.1. Study Design

To reveal young people’s perspectives on sexuality education, we chose a participatory approach. Working with young people as peer researchers allowed us to reveal the pupils’ perspective on classroom practices of sexuality education from an insiders’ perspective. The insider status of peer researchers increased access to study informants and empowered young people [35,36]. The literature suggests that peer researchers may establish a strong rapport with informants leading to a greater depth of the data and improved research of sensitive topics such as sexual health [35,37,38]. Studies show that with appropriate and adequate resources (time, financial investment), peer interviewing produces a positive, capacity building experience for peer-interviewers, participants, and researchers [35,38].

This study addresses four main research questions: (1) What does good sexuality education look like from the pupils’ point of view? (sub-questions: What content, what is needed to feel comfortable, when/by whom, and how should sexuality education be delivered?) (2) What do pupils value in the sexuality education they receive(d) during high school? (3) What elements are seen as problematic or missing? (4) Are there differences between pupils’ needs and wishes, related to differences in gender, sexual, cultural, and religious identities or level of education? As the peer researchers participating in our study were young and had not yet received any scientific education, we had to choose methods that fitted their skills. As inexperienced social researchers can lack skills in effective probing techniques, we asked the peer researchers to carry out short semi-structured interviews to explore the range of issues connected to how pupils experienced sexuality education at school. Subsequently, the issues raised were analyzed and explored more in-depth in Focus Group Discussions (FGD). FGD prove to be an effective method to explore the perceptions, ideas, opinions, and thoughts of participants [39]. As a third method, Photovoice sessions, were included to create a more open space for pupils to reflect on how sexuality education could be. Photovoice is a qualitative research method by which participants take photographs in response to a research question. The photographs are then analyzed by the same participants who work in small groups to identify common themes [40]. A review of the literature shows that Photovoice contributes to an enhanced understanding of community assets and needs and to the empowerment of participants [41]. Table 1 shows how the three methods contributed to the research questions and what questions were asked.

### 2.2. Recruitment of the Peer Researchers

In the preparation phase, we discussed with teachers and pupils how pupils could be motivated to join the research team. The best option was to offer the research project as an assignment in the last year of high school where each pupil had to conduct a small-scale study (‘Profielwerkstuk’ in Dutch). Pupils are free to choose their own subjects for this study. Our aim was to recruit around 20 peer researchers, working together in teams of three to four peer researchers per school. We recruited peer researchers by publishing a call for co-researchers on different websites aimed at high school pupils, young people in general and LGBTQ youth. We conducted the research with 17 peer researchers, aged between 16 and 18 years old, based at six schools, spread throughout the country. We strived to recruit peer researchers of different genders, sexual orientations, and cultural backgrounds, but succeeded only partly, with only four male and 13 female peer researchers, three peer researchers identifying as LGBTQ, and three people having a migrant background. Table 2 presents the gender and team division of the peer researchers who participated in this study. Three groups consisted of two peer researchers, one with three participants and two with four participants. The numbering of the schools corresponds with the schools in Table 3. 

As the schools were connected to the peer researchers, we did not explicitly select the schools participating in our research. Fortunately, the schools were in different parts of the Netherlands, in urban and rural environments, and located at different levels of education. Table 3 presents the school locations, education types, religious affiliation, composition of the school population, and sexuality education methods used per school.

### 2.3. Capacity Building and Coaching of Peer Researchers

Building upon the Explore toolkit developed by Rutgers and IPPF [42] on meaningful youth participation in research, two two-day capacity building trainings were organized during residential weekends. The first training was aimed to provide the peer researchers with enough tools to collect data at their schools. The first residential weekend also allowed the young researchers to meet each other, learn about the objectives of the research project, and reflect on their own norms and values regarding sexuality. The capacity training involved sessions on qualitative research techniques (how to conduct interviews and FGD, how to ask open questions) and on ethics. During the weekend, the peer researchers tested and adapted the interview topic list. After the weekend, a research supervisor was allocated to each team of peer researchers. This supervisor actively supported the peer researchers, gave feedback on their data collection, and arranged debriefings to discuss any difficulties. The supervisor also supported on a practical level by leading FGD and Photovoice assignments. The second two-day capacity training took place after the data collection and before the data was analyzed. The training was dedicated to a preliminary analysis of the research findings and a joint reflection on the research process and experiences of peer researchers. 

### 2.4. Data Collection

**Figure 1:**
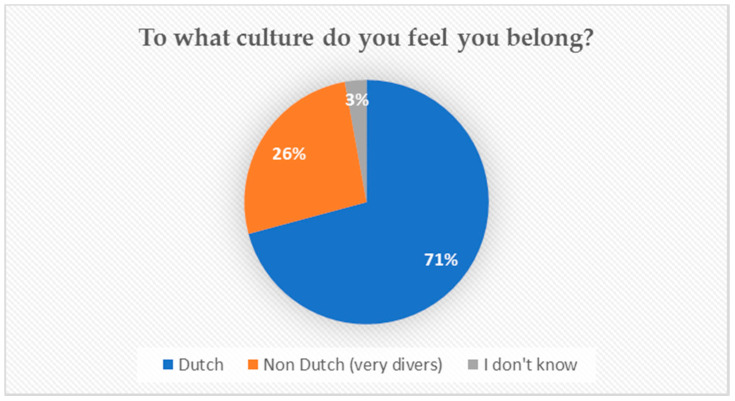
Cultural belonging participants.

The peer researchers recruited participants at their own high school. They were explicitly instructed to recruit pupils of all ages, levels of education, genders, sexual orientations, and cultural backgrounds. This resulted in a diverse group of 300 pupils, aged 12–18, at various levels of education (see Table 4). Participants were asked what cultural group they felt they belonged to (see Figure 1). Around a quarter considered themselves culturally as non-Dutch (i.e., having a migrant background and adhering to the cultural values associated with this background). For ethical reasons, the peer researchers were asked not to inquire about someone’s sexual orientation: This could potentially feel unsafe for the participants, and they might not have been ‘out’ in the school environment. As for the representation of diverse backgrounds, we succeeded in including a broad group of pupils in our research and believe the age and gender division in our sample reflect parts of the diversity of young Dutch people. However, we did include relatively many participants attending the middle and higher levels (theory-based) of Dutch secondary education.

The peer researchers started their data collection with short interviews. To ensure consistency of the collected data, we asked the peer researchers to fill out an online questionnaire in Google Forms, while carrying out the ten-minute interviews that they would directly submit after the interview. As such, we were able to monitor the progress made and the results obtained. They also recorded the interviews, which were transcribed afterward. The interviews turned out to be manageable for the peer researchers. However, some interviews were limited in-depth, which might have been caused by a lack of confidence of some peer researchers to use probing questions, but it may also reflect the discomfort of participants on being interviewed on this topic individually. After the interview phase, the peer researchers organized nine FGD, and in two schools also three Photovoice sessions. To promote social safety for participants, we worked with small, gender-specific groups (four to six participants). At one school, we also conducted an FGD with the members of the Gender and Sexuality Alliance (GSA), consisting of pupils striving to achieve a safe climate at school for people of all gender and sexual identities. The FGD and Photovoice sessions were led by senior researchers. After the data collection of the peer researchers, the senior researchers conducted another six Photovoice assignments at a seventh school, with a very multicultural school population, with the aim to increase the cultural diversity of our sample. All FGD and Photovoice sessions were recorded and transcribed. The FGD lasted between 30 and 60 min, and the Photovoice sessions between 60 and 120 min. These methods held a higher threshold for recruiting participants. Compared to the interviews, however, the participants of the FGD were more candid and shared personal experiences more openly, and data from FGD and Photovoice assignments transpired to be the most important source of information during analysis. 

### 2.5. Data Analysis

As data analysis is a delicate and lengthy process, only a partial analysis could be achieved by the peer researchers in the limited time they had available. After the second weekend, dedicated to the analysis and writing a research report, the peer researchers analyzed their own data and wrote their report on the needs and wishes of pupils of their own school. The senior researchers analyzed the data of all six schools and the data from the additional seventh school. For this analysis, the qualitative software MaxQDA (VERBI GmbH, Berlin, Germany) was used. The method used was a thematic analysis [43]. The first author analyzed the first ten transcripts and drafted a preliminary coding scheme. The second author examined the same ten transcripts using the codebook. The first and second authors then discussed discrepancies in their conceptualizations and amended the coding scheme accordingly. The authors then separately coded all the remaining transcripts. The codes were compared and sorted into new categories. These categories were assigned to themes. When complete, the authors met to resolve any discrepancies, so that all applied codes, categories, and themes were mutually agreed upon. 

**Figure 2:**
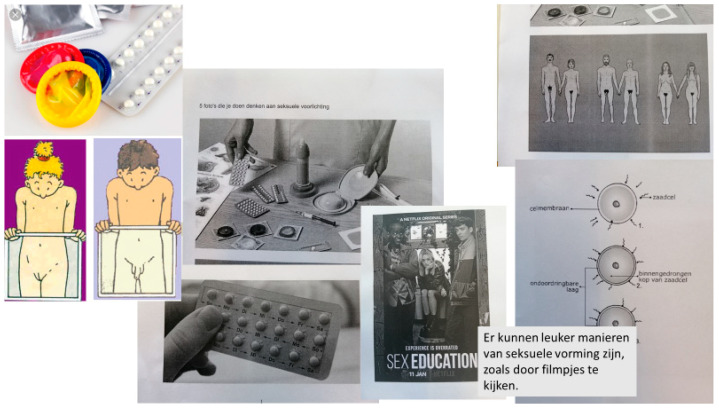
Photovoice pictures selection of a subgroup. (text saying: there are nicer ways of getting sexuality education, like watching movies).

**Figure 3:**
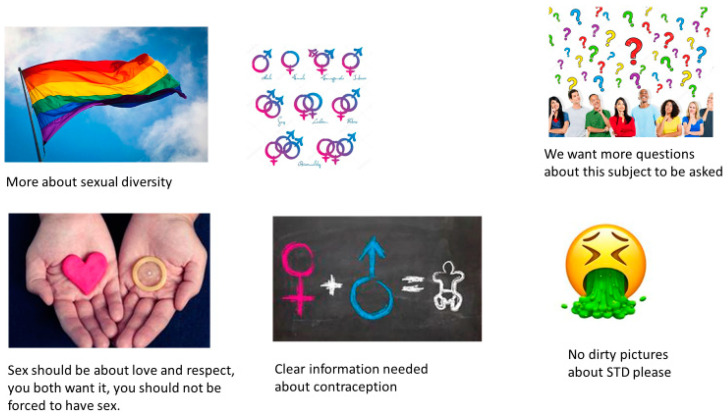
Photovoice pictures of another subgroup.

The interviews yielded codes on three main themes: Experienced importance of sexuality education; desired content of sexuality education; conditions for a safe atmosphere. The interviews gave a good impression of the perception of sexuality education to a wide range of pupils. The FGD generated much more details on how participants experienced current sexuality education practices, in what kind of situations they felt uncomfortable, what subjects they missed and how they felt sexuality education should be improved. This led to new codes under the same three main themes and added, for instance, much more codes on teacher skills under conditions for the safe atmosphere. Another main code was added, which was ‘methods to be used in sexuality education’. The discussions during the Photovoice sessions yielded mainly codes about the desired content of sexuality education and about methods. The Photovoice sessions added an extra source, which were visual images (see Figure 2 and Figure 3 for examples). Sometimes these pictures were plain photographs of contraception, condoms, a #MeToo poster, a rainbow flag, or a baby growing inside a belly, illustrating subjects that participants felt should be covered in sexuality education. In other cases, the explanation given by the participant revealed not just a subject, but another aspect of what she or he felt sexuality education should be about, like this girl explaining one of her pictures: *“This is a picture of a chair in an abortion clinic. Every woman must be able to make her own choice. That should be part of sexuality education too.”* The explanations are given and discussions in the Photovoice groups were coded in the same way as the FGD. 

### 2.6. Ethics

The research was conducted according to Dutch legal and ethical guidelines for responsible research, including voluntary participation, safeguards against participant identity disclosure, and respect for participants [44]. We explicitly asked the co-researchers and participants for their written consent, and naturally, participation was voluntary. Parent/guardian consent, however, was not necessary to obtain as the study was conducted within the school context, as part of the school curriculum. The confidentiality of participants was strictly safeguarded. To ensure that peer researchers could protect themselves and knew how to ensure the safety and confidentiality of their participants, we trained them on ethical principles during the first residential weekend. To illustrate, we taught the peer researchers how to create a safe atmosphere and find a place for the interview where nobody could listen in. They were also trained not to disclose any information and use only anonymized information in their report. Each group of peer researchers was assigned one of the professional researchers for support and guidance. Some studies reported special ethical challenges related to peer research [38]. Participating as a peer researcher can cause distress to the young people involved when they hear about problems or sexual trauma. Their involvement in a study on sexuality may also impact their social position. Bailey et al. [45] demonstrate in their systematic review that the benefits of participating for peer researchers outweigh the challenges, including greater confidence, new skills, and having their voice heard.

## 3. Results

### 3.1. Young People Express a Strong Need for More Sexuality Education during Their Whole School Career 

Most research participants voiced they want sexuality education at high school to be given more attention. The few participants who did not value sexuality education at school argued that they already know all there is to know, they do not need to know because they are not sexually active, or they feel too uncomfortable during sexuality education lessons. Clearly, younger participants (12–14 years old) had weaker ideas about sexuality education compared to participants aged 15 and older. *“Well, you should know about it, because it is part of life. So, it is useful to get some information.”* Many participants underlined that sexuality education prepares them to make good decisions in the future. *“So you know what to expect, what are sensible choices and what are not.”* Others stressed that sexuality education is relevant for their lives at this moment, as relationships and sexuality are very important issues to them. They emphasized the importance of continuity in sexuality education when they grow older and have more sexual experiences. *“I have the idea our teachers think you should know it all when you are 16. But that is not true for everyone. If you are 16, you don’t know all the implications. They throw you in at the deep end. Go discover it yourself, we won’t help you anymore.*” Most participants indicated sexuality education should be given throughout their school careers, starting from elementary school. “*By repeating more often and more frequently, it becomes a more normal thing to talk about from childhood onwards*.” Other reasons why they found sex education at school important, is that not everybody gets good information at home, and some participants argued that it’s hard to find proper and reliable information on the internet. And proper information is needed to empower young people to make their own decisions: “*There are families where contraception is taboo. But well, you should be able to think for yourself, without your mother deciding everything for you.”* Other studies among young people conform to a strong need for school-based sexuality education [30,31,32]. However, the specific demand to extend sexuality education to senior classes does not explicitly emerge from other studies, probably because it is more common in other countries that sexuality education is (still) taught to young people aged over 15 years.

### 3.2. Sexuality Education Should Cover More Issues 

Although almost every participant received some information about sexuality at school, this information in most cases only concerning contraception, reproduction, and STDs/HIV, usually provided in biology. Participants argued they want more sex-positive education. “*Now it is quite negative, while sex can be good too. They just tell you half the story. When nobody tells you how it should be, how can you recognize being in a bad relationship? So, I feel both sides have to be told.”* Female pleasure should be getting more attention, as a girl clarifies: “*It is important that sexuality education makes clear that girls need to have pleasure too, so boys do not feel sex is just for them. Boys need to know about vaginas, you know, about the most sensitive part, the clitoris.”* Participants particularly missed education about subjects that are relevant for them at present, not in the future, about feelings, relationships, dating, sexual harassment, communication, and online and offline sexual behavior. “*Sexuality education is focused too much on ovaries and too little on how it feels, or what you like. Or on how do you talk about sex together?”* Participants also missed information on how to deal with problems and conflicts: where to get a Sexually Transmitted Infection test, how does a pregnancy test work, where to turn to when you need an abortion, how to find help after sexual violence? They wanted concrete scripts for situations they encounter: “*When you do not want to have sex… How do you say that? And how can you be clear you want to move forward?”*

Both girls and boys disclosed that gendered social norms influence their self-esteem and their sexual agency. Girls protested the double standard and want to have this addressed in sexuality education. *“I think it is so wrong… the difference people make between girls and boys… when a girl has sex once, she is a slut, but when a boy has sex ten times, he is the hero for all the boys..“* In one of the focus groups, boys discussed the shaming and blaming of girls sending sexy selfies: *“I do understand why some girls are called a slut, if you see the selfies they post.. they just want attention.”* Another boy responded: *“If a boy sends a dickpic, do you think he is a whore too?”* The answer of the group: “*No, no, he is just a fool.*” Another area where gendered norms play out are uncertainties about their bodies. Participants argue sexuality education can play a role in diminishing uncertainties: “*Many boys and girls are insecure about things that are very normal. It is important to talk about this, to know that it is not crazy. That sex is not shameful. Relationships are also important to talk about. Just to break the taboo.”* Participants also emphasized that it is important to know that everybody develops physically and emotionally at their own pace. “*So, you don’t need to be ashamed if you are late having sex or getting your period. We need more diversified examples. Different stories. To learn that development is not one-dimensional. Everybody is different.”* Moreover, especially girls wanted to know more about their rights: “*It is good to know when you are entitled to say no, to know your rights, especially around #MeToo. When can you stop and say ‘this is it! These are my rights. I do not want this!’”*

When it comes to sexual and gender diversity, many participants reported that it is often dealt with in a very limited way. “*Yes, they do admit it exists, but we don’t talk about it. We don’t really address it. I feel it should be dealt with differently. Not just mentioning that bisexual, gay, and transgender people exist, but also mention heterosexuality (…) So not to pretend heterosexuality is the norm. Just talk about all sexualities.*” Participants wanted sexual diversity integrated into the whole area of sexuality education, not isolated as a single issue. They want to have a more comprehensive approach to sexuality education, including subjects like sexual orientation and gender identity, consent and coercion, online sexual behavior and sexual pleasure. This is in line with findings in other countries of the needs of young people regarding the content of school-based sexuality education [10,11,12,13,14,15]. Studies also confirm that current Dutch sexuality education fails to address the social norms that limit young people in their sexual development and relationships and reproduce inequalities [17,28].

### 3.3. Sexuality Education Requires a Safe Atmosphere and a Self-Confident and Sensitive Teacher

According to the participants in our study, sexuality education can only succeed if there is a safe atmosphere in the classroom. If they felt uncomfortable, they would not talk about sexuality. *“Just a nice atmosphere in which you can feel comfortable. So, you don’t have to be afraid to show who you are. That you can just be yourself.”* Participants thought differently about sharing personal experiences. Some participants did not want to share anything personal, while others very much want to. “*I do want sexuality education, but if you have no questions or you don’t want to talk in a small group, I feel it should be not mandatory, you know like ‘you have to talk about it’*.“ Participants shared stories about friends being bullied after sharing personal information. *“Of course people are allowed to make jokes, but not nasty jokes… like they do about homosexuality or racist jokes…”* Participants argued they need teachers to be sensitive to this risk of exposure and bullying and not to expect them to open up in front of a whole class about their own experiences. The possibility to ask questions anonymously, working with available stories (in films, or written), and working in small groups helped to increase safety.

Many pupils liked their own teacher (biology, social science, or mentor) to teach sexuality education as they were familiar with him or her. Some participants preferred having a different expert teaching sexuality education because it felt too intimate to share sexual content with their own teacher. Ultimately, it depended on the competence of the teachers. Participants mentioned four competencies of teachers that are crucial. First, teachers should be at ease with giving sexuality education. “*A teacher should be self-confident in teaching sexuality education. Not someone who hesitates all the time and obviously feels shy. Then I think ‘never mind’…*” Second, teachers should encourage young people to form their own judgment. For instance, when discussing porn, a participant warned about moral judgments: “*Of course, porn is exaggerated. But you must be careful not to pressure people how sex ought to be. That is up to the person themselves. Maybe there are women who like anal sex, who knows? So, it is not right if a teacher says that porn is not realistic, and women do not want to be treated that way. It makes no sense to start about different sexualities, hetero, gay, bi, to make people accept each other, and then continue that certain forms of sex are not OK. What you should say is that porn gives an idealized image of breasts, vulva, large penis, that does not match reality.”* Third, teachers should take young people and sexuality education seriously. Participants explained that a teacher must find a delicate balance between seriousness and humor. *“The atmosphere should not be too tense, because nobody will interact, but it should also not be too treated like a laughing matter, because people will not feel safe to open up.”* Fourth, pupils want a teacher they can trust, someone they can turn to if something bothers them. It does not matter to most participants whether the teacher is young or old, a man or a woman. Mentors were often referred to as good teachers, because they are a little closer to young people. The finding that it is not the gender or age of the teacher that matters, but their competence to talk openly, be trustworthy, and facilitate meaningful discussion, corresponds with findings by other scholars [11,12]. One of the main issues Pound, Langford, and Campbell [31] found was that young people felt sexuality was dealt with too casually. This need for sensitivity to the ‘specialness’ of sexuality is mirrored in the urge for a safe atmosphere. However, unlike the young people in the ten countries included in the meta-analysis of Pound, Langford, and Campbell [31], Dutch young people obviously do not feel that teachers neglect or deny the fact that they are sexually active. Probably this is connected to the Dutch dominant cultural logic, which accepts adolescent sexuality [2]. In fact, young people voiced that the lack of sexuality education in senior grades gave the impression that they were supposed to know everything at the age of 16 and be self-sufficient and agentic in their sexual experiences. 

### 3.4. Young People Want Diverse Teaching Methods 

Participants expressed their need for diverse sexuality education teaching methods in the classroom: Discussions, practical assignments, tips, movies, and games. They did not want to listen to lengthy explanations from their teacher but wanted to work through more interactive methods. *“For instance about STDs, you can also make small groups and each group gets the assignment to explain about one STD, how can you get infected, is it a bacterium or a virus, how is it treated, what are the consequences,* etc. *Then you get more interaction between pupils and not just your teacher talking all the time.”* Participants had different ideas about splitting the class into separate boys’ and girls’ groups. Some argued it would create more safety to ask delicate questions when you have separate boys’ and girls’ groups, where others felt it should be mixed so you can learn about the other sex as well. *“Just together, because you also have to know how the other sex feels about things. Recently there was a boy who asked me, how many times do you have your period, four times a year? I thought ‘really!’”* Participants also stressed the importance of hearing people with different opinions. “*It is good to get out of your comfort zone and hear people who are looking from another perspective, to understand their views.*” According to our participants, guest speakers and ‘experience experts’ are a good addition to the lessons of a teacher. *“Especially about things that are not accepted by society. Then you have a very quick tendency to judge because you feel it’s not normal. But if there is a guest speaker who has experienced it themselves, then you know someone, and you feel much more respect for such a person.”* Participants wanted variation in the lessons and methods, to get the opportunity to form their own ideas and points of view on socially sensitive topics, such as abortion, sexting, and sexual violence. This finding corresponds with the recommendations by other scholars to provide multiple stories in sexuality education, to enable young people to form their own views and identities and learn to navigate conflicts [30,46,47]. Naezer [47] (p. 720) concludes from her study of Dutch youth, ‘Finding people who are able and willing to confirm the “normalcy” of certain feelings, experiences and identifications requires the availability of a multitude of perspectives, a requirement that is absent from Dutch sex educational policies.’ 

## 4. Discussion

In this section, we will reflect on the main findings and make suggestions for the steps to be taken to realize the changes in sexuality education desired by young people. Firstly, Dutch youth participating in our study clearly voice the need for more lessons of sexuality education, delivered throughout their whole school careers, starting from elementary school and continuing as they grow older and start having romantic relationships and sexual experiences. This is a clear message to developers of school curricula and teachers to expand the amount of time spent on sexuality education and ensure that it does not stop when pupils are 15 years old. Currently, the debate about sexuality education in the Netherlands has a strong focus on what age to start with sexuality education. Less attention is paid to the age at which to end sexuality education. Our study underlines the importance of continuing sexuality education after the age of 15.

Secondly, our study clearly illuminates the need for a truly comprehensive and sex-positive sexuality education. Young people miss information on sexual consent and sexual coercion, sexual diversity, sexual pleasure, relationships, dating, online and offline communication, and sex in the media. “*Sex is more than biology*”, as one of our participants voiced. Although this has been voiced by many young people over the past years, the sexuality education that is taught at many Dutch schools still has a very limited scope. A possible way forward could be to integrate comprehensive sexuality education into a broader curriculum than biology, for instance, in civic education, social sciences, or ‘citizenship competences’, as a relatively new course taught in Dutch schools. Citizenship competences are defined as the knowledge, skills, attitudes, and reflection needed by young people to fulfill social tasks that are part of daily life in a democratic and multicultural society [48]. This study demonstrates that many young people experience tension, which arises from the collision between their needs and feelings and what they perceive as social norms regarding good decision-making, heterosexuality, and gender roles. Sexuality education will benefit from broadening the scope beyond lessons on individual decision-making to address norms collectively and offer learning opportunities and reflectivity [46,49].

Thirdly, one of the most crucial conditions for good sexuality education, is a safe class atmosphere, which enables young people to feel comfortable and relates to the issues presented. This means teachers must be aware and sensitive regarding the social dynamics going on during sexuality education, and outside of the classroom. Teachers should be aware that sexuality is enacted through collective practices, which brings about social effects of popularity, ethnicity, and gender [28]. For instance, by being aware of the kind of questions that can be asked publicly, and the ones that cannot, by giving space for anonymous questions, by carefully structuring lessons, and by using methods that support social safety. A safe space is especially important for young people fearing social stigma or judgments, like LGBTQ youth. As teachers are also immersed in a dominant heteronormative culture, they need to reflect on their own assumptions and prejudices. Research indicates that teachers might unintendedly reinforce stereotypes and contribute to inequality [28,50]. Methods used to address homophobia may be contra-productive when LGBTQ people are positioned as ‘different’ and vulnerable, and by doing so, could reinforce the presumption of heterosexuality. Classroom discussions of homosexuality might encourage controversy [51] and can even initiate hate speech [52]. Teachers need education and training to update their knowledge on young people’s varying sexual identities, relationships, and sexual cultures. They need to be aware of various ways of promoting inclusivity, such as by using inclusive terminology (for instance, using partner instead of boy- or girlfriend, avoiding using sex synonymously with sexual intercourse) and resources that represent sexual diversities [29].

Fourthly, the need for diversity and interactivity in teaching methods requires an innovative approach. In its Guidance, UNESCO promotes a learner-centered approach to sexuality education and encourages collaborative learning strategies [53]. When sexuality education aims to empower youth, rather than diminish health risks, teaching methods will become an important object of scrutiny [54]. Empowering methods need to put young people at the center and be sensitive to (the heterogeneity of) their concerns, realities, suggestions, interests, and resistance [55]. Moreover, methods should include supporting young people’s own ways of knowledge building and learning strategies [56]. Developing sexuality education that builds on young peoples’ active participation will ensure content that is much more relevant to their lives, and additionally, it will always be up to date in language, dating trends, and technological developments. 

This study has some limitations. The main limitation is that we were unable to explore whether differences between pupils’ needs and wishes were related to differences in sexual, gender, cultural, and religious identities. As we did not gather much information on the sexual orientation and ethnic background of participants, we cannot make proper distinctions based on these categories within our group. Ethically, this was information that could not be obtained using face-to-face research methods among pupils in a school context. A recommendation for future research would be to combine face-to-face methods with more anonymous data collection methods. 

## 5. Conclusions

This study explored what sexuality education should be like, from the perspective of Dutch young people, aged 12–18. Seventeen young peer researchers collected data at their own high school, among 300 participants. Our study endorses that the benefits of participatory research outweigh the challenges [45] both for the quality and reach of the study as for the development of the peer researchers. The findings of our study demonstrate that most young people want more lessons, and more comprehensive sexuality education than they currently receive. They want sexuality education to move beyond biology and focus on matters that are relevant in their actual lives, such as dating, online behavior, sexual pleasure, relationships, and sexual coercion. Moreover, they want sexual diversity integrated and normalized in all sexuality education content, instead of treated as a separate issue. One of their main issues is that sexuality education should be taught in a safe class atmosphere, which requires a teacher who is sensitive enough to know which questions can be answered publicly, and which cannot, and who takes young people seriously and encourages them to form their own judgments. The future challenge for developers of sexuality education and teachers is to move away from considering the teacher as an expert transferring knowledge towards a participatory, learner-centered approach. The role of the teacher will change into a facilitator of learning and empowerment by encouraging young people to discover different sexual cultures and identities, exchange knowledge, and take a stance. An active role of young people will ensure that the content of sexuality education is more relevant to young peoples’ lives and provide more opportunities to develop their sexual agency. 

## Figures and Tables

**Table 1:** Aims and questions per research method.

Method	Aim	Questions
Semi-structured Interviews	Contributes to research question 2, 3, and 4 by exploring: The level of felt importance of sexuality education; The aspects pupils are satisfied with and the aspects they find problematic in the sexuality education they received.	Do you think it is important that you are taught about relationships and sexuality? Have you already had sexuality education in high school? When did you have sexuality education in high school? What was it about? Are those the things it should be about? Did you miss things? What did you like about the lesson(s)? What would you change about the lesson(s)?
Focus Group Discussions	Contributes to research question 1, 2, 3, and 4 by exploring more in-depth: Whether they feel sexuality education is relevant and important to young people; The experiences of participants with sexuality education at school; The topics pupils find important to be addressed; When, by whom, and by which methods sexuality education should be taught; The conditions that are needed to create a safe atmosphere.	How do you experience sexuality education at school? Is the content of sexuality education relevant to your life? What makes it relevant, and what doesn’t? What topics do you find important in sexuality education? How should sexuality education lessons be taught? What conditions are needed to make you feel safe when you are taught about relationships and sex? In which grade(s) do you think sexuality education should be given? Who should be teaching sexuality education?
Photovoice Sessions	Contributes to research question 1 by exploring in a visual way: What good sexuality education looks like, in the eyes of pupils	What does good sexuality education look like?

**Table 2:** Gender and division in teams of peer researchers (*n* = 17).

School	Girls (*n* = 13)	Boys (*n* = 4)	Other (*n* = 0)	Total (*n* = 17)
1	3			3
2	2			2
3	2			2
4	2	2		4
5	2	2		4
6	2			2

**Table 3:** School locations, education types, religious affiliation, the composition of the school population, and sexuality education programs.

School	Location	Education Type	Religious Affiliation	Composition School Population	Sex Education Methods
1	Boxtel (rural)	HAVO/VWO (middle and higher level)	Roman Catholic	Mainly white	Biology textbooks
2	Rotterdam (urban)	Gymnasium (Higher level)	Christian	Mainly white	Biology textbooks
3	Hengelo (rural)	HAVO/VWO (middle and higher level)	Public	Mainly white	Biology textbooks
4	Almere (urban)	VMBO/HAVO/VWO (all levels)	Christian	Multicultural	Biology textbooks
5	Althorn (rural)	HAVO/VWO (middle and higher level)	Roman Catholic	Multicultural	Biology textbooks, Gender and Sexuality Alliance
6	Breda (urban)	VMBO/HAVO/VWO (all levels)	Public	Multicultural	Biology textbooks; method Long Live Love

**Table 4:** Participant characteristics (*n* = 300).

Participants.	Girls (*n* = 156)		Boys (*n* = 144)		Other (*n* = 0)
	Educational Level		Educational Level		
	Practice-Based	Theory-Based	Practice-Based	Theory-Based	Total
Peer researchers	0	13	0	4	17
Participants interviews	26	71	32	67	196
Participants FGD	3	23	8	8	42
Photovoice participants	8	12	12	13	45
